# Effectiveness and therapeutic alliance between face-to-face and online psychological interventions. A longitudinal study

**DOI:** 10.3389/fpsyg.2025.1624438

**Published:** 2025-10-28

**Authors:** Josep Mercadal, Laia Coromina, Victor Cabré

**Affiliations:** ^1^Vidal i Barraquer Foundation, Barcelona, Spain; ^2^Institut Universitari de Salut Mental Vidal i Barraquer, Barcelona, Spain

**Keywords:** effectiveness, online psychotherapy, face-to-face, mental health, therapeutic alliance

## Abstract

**Background:**

Since the pandemic, there has been an evident increase in demand for online psychotherapy. There exist studies focusing on the effectiveness of online therapy and identifying the situations in which it may be helpful, but a gap in literature was found on studying the effectiveness and therapeutic alliance of online psychotherapy compared to face-to-face psychotherapy.

**Objective:**

The aim of this study is to evaluate the effectiveness and the evolution of therapeutic alliance between face-to-face and online psychological interventions, from the perspective of both therapists and patients. This article aims to be a continuation of the study initiated by Mercadal and Cabré in which, among other conclusions, it was found that the therapeutic alliance in an online intervention was significantly good, although not as good as in a face-to-face intervention.

**Methods:**

A total of 187 subjects aged between 18 and 29 years old participated anonymously and voluntarily in the study, 81 (43.3%), of whom were men and 106 (56.7%) were women. The instruments used were socio-demographic data, the patient version of SOFTA-o (System for Observing Family Therapeutic Alliances-observational), CORE-OM (Clinical Outcomes in Routine Evaluation-Outcome Measure), and HoNOS (The Health of the Nation Outcome Scales).

**Results:**

The results show that there is more preference for the in-person modality than online. A correlation is also observed between CORE-Om pre and HoNOS pre; and CORE-OM post and HoNOS post, which indicates agreement between patient and therapist regarding the evolution of the treatment. At the same time, patients and therapists report better results in person than online (*d* = 0.76 and *d* = 0.91, respectively).

**Conclusion:**

Therapists perceive a greater improvement after the treatment rather than do patients. In addition, post-treatment scores showing an improvement in the symptomatology are related to a greater Therapeutic Alliance after treatment. Concerning the main aim of this article, both patients and therapists reported that face-to-face therapy obtains better results than the online modality, a finding consistent with the authors’ preliminary studies. However, there are some limitations, such as self-selection of modality by participants, the use of a single therapist, the sample of university students, and the lack of post-intervention follow-up.

## Introduction

### Background

Ever since online psychotherapy was conceived as an alternative to ordinary therapy, especially during lockdown, there has been an evident increase in demand for online psychotherapy.

As Bauman (2005) introduced, we live in a liquid society which means that we are constantly adapting to the new stimuli we face. In the same liquid way, we can either choose to adapt and move forward or remain in conventional patterns. The most recent event occurring in our society to which we had to adapt is the digital revolution. Modern day lives revolve around electronic devices, so naturally the concept of online therapy comes as no surprise to the new generations. There are currently four generations living in the same decade, so their opinions tend to differ when it comes to the question if online psychotherapy offers the same depth as a face-to-face method. In a recent study, Yılmaz et al. (2024) found that opinions about online psychotherapy differ between young and middle-aged patients, with young people being more inclined towards online psychotherapy.

The current society is ruled by a harmonious fusion between immediacy and comfort.

It is also interesting to talk about how relationships have also changed in recent years. In a liquid society there are, needless to say, liquid relationships. We have come to a place where relationships are shaped by the values of our current society: immediacy and comfort. We cannot define modern day relationships without the concept of “digital.” This digital component has brought us closer in some aspects and more distant in others. People expect to connect with therapists in the same way as they do in personal relationships, introducing a screen between the two people which, unironically, can seem to bring them closer (Mercadal and Cabré, 2021).

Culture of immediacy is rapidly overtaking our society and especially the newest generations, who prefer digital over physical. It is easy to assume that they will also be more inclined towards online psychotherapy, which can explain the higher demands in online psychotherapy nowadays.

### Psychological intervention

Online psychotherapy has been considered a complement to traditional face-to-face psychotherapy (Berle et al., 2015; Cipolletta et al., 2018; Norwood et al., 2018), but it has been shown to be a valid alternative in a number of studies when face-to-face psychotherapy is not possible (Cook and Doyle, 2002; Knaevelsrud and Maercker, 2006; Reynolds et al., 2006; Preschl et al., 2011; Wagner et al., 2014; Holmes and Foster, 2012; Lewis et al., 2021).

Since the late 1990s, research into online psychotherapy has focused on its effectiveness rather than knowing in which situations it might be helpful (Stoll et al., 2020). However, it was found that online therapy can be helpful in people who have difficulty asking for help, as it can provide a sense of shelter and protection (Schultze, 2006; Tate and Zabinski, 2004; Vallejo and Jordán, 2007), unless there is a hidden reason behind the client’s proposal to start online therapy, such as keeping distance or feeling more protected, because behind this request there could be relational problems that prevent them from coming (Cabré and Mercadal, 2016). Nevertheless, it is not recommended for patients with a lack of emotional control, such as psychosis, major depression, violence or in crisis situations (Torre and Pardo, 2019), as dealing with crisis and suicidality could be challenging in online psychotherapy. However, in patients diagnosed with depression and anxiety, combining face to face with online therapy was found to be more effective than just face to face therapy (Zwerenz et al., 2017).

Bibliography differs from opinions as some authors think that it is the therapist’s style and personality that determine the therapeutic relationship (Cipolletta et al., 2018) and therapy effectiveness (Rathenau et al., 2021) rather than the modality of the psychotherapy. As for outcomes in different psychotherapeutic models, Rosenzweig (1936) introduced the idea that all therapies work through common factors, meaning that different therapies produce similar outcomes (Budd and Hughes, 2009; Luborsky et al., 2002; Marcus et al., 2014; Cuijpers et al., 2018). Building on this concept, Lambert (1992) pie chart model and the study by Cuijpers et al. (2012), suggested that approximately 30% of therapeutic change can be attributed to common factors, 15% to specific techniques, 40% to additional therapeutic factors and 15% to the placebo effect. Key common factors include therapeutic alliance, therapist empathy, and client expectations (Browne et al., 2021). Horvath et al. (2011) proved that stronger therapeutic alliances are associated with better treatment outcomes. However, the evidence is correlational and cannot establish causality (Norcross and Wampold, 2011).

While therapeutic alliance is a common factor, it also plays a different role in each psychotherapy model. In therapies which emphasize therapeutic alliance, such as psychodynamic therapy, the alliance may be more linked to the outcome than in therapies that do not emphasize the therapeutic alliance, such as Cognitive Behavioral Therapy (CBT) (Huibers and Cuijpers, 2015; Romero-Moreno et al., 2023). Nevertheless, online CBT has been found to be the most effective, as it is also the most studied one (Weightman, 2020). Although, more research is needed to determine the online effectiveness of other types of psychotherapy such as psychodynamic (Carrillo et al., 2021; de Bitencourt Machado et al., 2016; McDonald et al., 2020).

Despite the proven relevance of common factors for effective psychotherapy, especially those of an emotional nature, Romero-Moreno et al. (2021) found that therapists still have doubts about the factors responsible for psychotherapeutic effectiveness. They do not place particular value on specific factors, such as the techniques used or the therapeutic approach adopted.

According to Romero-Moreno et al. (2021, 2023), therapists’ perspectives on the importance of emotional factors, including the therapeutic alliance, are shaped by their theoretical approach and their views on the relative effectiveness of different psychotherapy models. Hence psychodynamic therapists place greater importance on the therapeutic alliance, cognitive behavioral therapists emphasize directiveness and support, and eclectic therapists highlight common and emotional factors for therapeutic change.

The benefits of online psychotherapy found in the recent bibliography are that online sessions can reduce therapist’s workload and improve access to healthcare, particularly in rural areas (Stoll and Trachsek, 2019) whilst maintaining the quality of care (Carrillo et al., 2021). It can provide flexibility for both therapists and patients as it can be delivered in remote locations (Lippke et al., 2021).

Some of the challenges of online psychotherapy are actually reflected in the setting, as clients may be in the same environment as the people with whom they are struggling. Online therapy can overly condition the intimacy of the session and posteriorly the development of the treatment (Mercadal and Cabré, 2021). Another aspect altered in the setting is that online psychotherapy lacks a “frame” (Ashraf et al., 2020), which can provide a secure base and a reassuring effect whereby ‘even chaos, once it has been given a name, is less chaotic’ (Milner, 1950). The travel time also constitutes part of the setting, and it can give both patients and therapists the opportunity to reflect about the content of these appointments (Sayers, 2021).

Overall, these issues may explain the high dropout rates in online psychotherapy (Boldrini et al., 2020). Some other factors that should be addressed and may contribute to dropout are women, younger patients, unpartnered patients, less educated patients, patients with depressive symptoms and those with fewer expectations of therapy (Lippke et al., 2021).

Videoconferencing is an interaction with a great amount of sensory components (Cabré and Mercadal, 2016), and a common experience amongst therapists is that they feel a greater fatigue experienced with online activity (Mercadal and Cabré, 2021), also known as “zoom fatigue” due to excessive use of videoconferencing platforms, especially during the pandemic (Bennett et al., 2021; Epstein, 2020). In addition, therapists reported feeling a sense of loss of presence and engagement in the online mode (Gullo et al., 2022). Results consistent with Malouin-Lachance et al. (2025), who concluded that, while digital interventions improve accessibility and engagement in mental healthcare, they also present challenges related to limited emotional depth, personalization, and ethical considerations.

Yılmaz et al. (2024) used qualitative research to assess how patients perceived both methods. The most frequent metaphors about online psychotherapy were associated with the categories of convenience, artificiality, similarity to face-to-face psychotherapy, and ineffectiveness. And the metaphors about face-to-face psychotherapy belonged to the categories of contact, effectiveness, reality, and difficulty. Recently, Leuchtenberg et al. (2022) found that patients and psychotherapists mainly preferred face to face psychotherapy.

Ierardi et al. (2022) studied the “Effectiveness of an online versus face-to-face psychodynamic counseling intervention for university students before and during the COVID-19 period.” The aim of the study is to evaluate the effectiveness of the online counseling intervention during the COVID-19 pandemic, compared to face-to-face interventions. The results show that the online counseling intervention during the pandemic was effective in reducing psychological distress scales such as depression (*p* = 0.008), obsessive-compulsive (*p* = 0.008), interpersonal sensitivity (*p* = 0.005), and anxiety (*p* = 0.011), as well as the total scale of the SCL-90 R (*p* = 0.017). The face-to-face counseling intervention was effective in reducing psychological distress in all subscales and in the total scale of the SCL-90 R (*p* = 0.000) and in increasing the level of life satisfaction (*p* = 0.023). Attachment style did not influence the effectiveness of the online and face-to-face interventions. However, face-to-face interventions were more effective than online therapy in reducing a wider spectrum of psychopathological problems and in increasing life satisfaction.

To date, there is a gap in literature on the effectiveness of online psychotherapy compared to face-to-face psychotherapy. So far, it has been found to be effective (Barker and Barker, 2022; García et al., 2022), although online therapy does not provide any additional benefit when compared with to face to face therapy (Rollman et al., 2018). However, face-to-face interventions have proven to be more effective than online therapy in reducing a wider spectrum of psychopathological problems and in increasing life satisfaction (Ierardi et al., 2022).

As there is still some uncertainty about the impact of online therapy, before simply opting for an online format, Carrillo et al. (2021) suggest that we should consider which patients are most likely to benefit from a face-to-face format. Although online therapy can be delivered to almost all patients, not all patients are suitable for this modality (Cabré and Mercadal, 2016).

### Therapeutic alliance: online vs. face-to-face

Essential aspects of psychotherapy are the therapeutic relationship and alliance, consistently ranked among the most reliable predictors of positive therapeutic change. They are regarded as a one of the most consistent predictors of effective therapeutic change and constitute a central focus across all major psychotherapy models (Norcross and Lambert, 2011, 2019; Flückiger et al., 2018; Wampold and Flückiger, 2023; Karunarathna and Jayawardana, 2024; Opland and Torrico, 2024; Malouin-Lachance et al., 2025). This is further supported by evidence indicating stronger alliances, characterized by a solid bond between therapist and patient, as well as agreement on therapeutic goals and tasks, have been found to be associated with better patient outcomes (Horvath et al., 2011; Wampold, 2015).

In recent studies, patients rated the therapeutic alliance higher than psychotherapists, a result consistent with previous studies (Lopez et al., 2019; Simpson and Reid, 2014), and therapists rated the therapeutic success nearly the same as patients (Eichenberg et al., 2022). Another study found out that while patients reported equivalence in face-to-face and online psychotherapy, therapists experienced an equivalent bond but more advantages in tasks and goals in face-to-face therapy (Leuchtenberg et al., 2022).

Face-to-face human treatment is not comparable to online therapy, although sometimes it can be a good resource, in no case it is more real than face-to-face interactions (Mercadal and Cabré, 2022). Body language, facial expression and the pheromones are crucial to establishing human relationships, and they are aspects missing in online psychotherapy (Lemma, 2017; Cheshire et al., 2020), which influence the empathy perceived by the patients. However, if therapists have previously seen the patient face-to-face, it can be easier for them to interpret their gestures, expressions, silences or pauses through the screen, which explains why psychotherapists still experience a comparable bond with patients (Leuchtenberg et al., 2022).

Empathy is a reasonably strong predictor of therapy outcome (Elliott et al., 2018), meaning that it can also determine an adequate working alliance between psychotherapist and patient, regardless of the psychotherapeutic approach (Elliott et al., 2011). The importance of empathy in the patient-psychotherapist relationship is widely recognized in literature (Feller and Cottone, 2003; Nascivera et al., 2018).

Sperandeo et al. (2021) studied whether if empathy worked the same way during online sessions. Their results suggest that therapists perceived themselves as being equally capable of providing empathy and support in both settings. Another finding was that the therapists who liked online interventions were mainly those who had previously used this technique. Patients, on the other hand, felt that psychotherapists are more empathetic and more capable of providing support in the online sessions. Due to the pandemic, we must take into account the particular situation in which face-to-face sessions did not offer the same level of comfort as online sessions. At that time, in-person sessions were carried out with masks, plexiglass dividers and strict social distancing. Patients felt much more understood in this setting because they could perceive facial expressions and voice intonations (Sperandeo et al., 2021).

The mixed results regarding the therapeutic alliance may mean that the patient-psychotherapist relationship in online psychotherapy might not yet be optimal (Leuchtenberg et al., 2022). This issue could be addressed with specific training programs for therapists to adapt psychotherapy methods to digital settings, improve the management of technical issues or service evaluation (Leuchtenberg et al., 2022; Lippke et al., 2021; Carrillo et al., 2021). It could help to improve online therapy and make it an effective treatment option to complement face to face therapy, not as a substitute, and also improve the provision of mental health services if we ever need to be prepared to face a situation similar to COVID-19 (Leuchtenberg et al., 2022; Longobardi et al., 2018).

This longitudinal study aims to evaluate the evolution of the therapeutic alliance and face-to-face and online psychological interventions. In this sense, we follow the hypothesis that the face-to-face modality will present a better therapeutic alliance and better patient evolution (both from the patient’s and the therapist’s point of view) between before and after the intervention, compared to the online modality.

## Methods

### Participants

A total of 187 subjects participated anonymously and voluntarily in the study, 81 (43.3%) of whom were men and the resting 106 (56.7%) were women. The subjects were aged between 18 and 29 years old, with a mean age of 23.01 (SD 2.82; Table 1).

**Table 1:** Descriptive results.

Characteristics	Values
Age (years), mean (SD; range)	23.01 (2.83; 18–29)
Pre CORE-OM scores, mean (SD; range)	108 (11.32; 80–130)
Post CORE-OM scores, mean (SD; range)	75.7 (11.67; 52–111)
Pre HoNOS scores, mean (SD; range)	39.65 (4.17; 31–47)
Post HoNOS scores, mean (SD; range)	22.69 (7.16; 11–39)
Pre TA scores, mean (SD; range)	8.56 (2.97; 3–18)
Post TA scores, mean (SD; range)	36.11 (13.95; 11–56)
Gender, *n* (%)	
Male	81 (43.3)
Female	106 (56.7)
Modality, *n* (%)	
Web-based	86 (46)
Face-to-face	101 (54)
Diagnosis, *n* (%)	
Anxiety	58 (30.0)
Depression	70 (10.7)
Grief	15 (8.0)
Mistreatment	28 (15.0)
Family problems	11 (5.9)
Couple problems	11 (5.9)
Concentration problems	9 (4.8)
Social relation problems	17 (9.1)
Adaptation problems	15 (8.0)
Others	3 (1.6)

Initially, a sample of 202 subjects was included, although this was reduced to 187 since 15 of them did not complete the treatment (11 in online treatment and 4 in face-to-face treatment).

The participants came voluntarily and free of charge to the psychological guidance and counseling service, from two universities in Barcelona. At this service, they received counseling and those who were indicated to start a psychological treatment were invited to participate in the study.

### Instruments

The participants responded to the following questionnaires: 1. Sociodemographic data: sociodemographic data such as age, gender, treatment modality (online or face-to-face), and diagnosis were collected *ad hoc*; 2. Therapeutic alliance—SOFTA-o (System for Observing Family Therapeutic Alliances—observational) for patients (Friedlander et al., 2001); this instrument was created simultaneously in English and Spanish as a transtheoretical tool for research and practice in TA. In this study, the patient version was used. The measure is based on three dimensions: engagement in the process, emotional connection, and safety. It also provides an overall score. The 12 items, both negative and positive, are related to patients’ behaviors, which are grouped within the three dimensions; 3. Clinical Symptomology: The Spanish version (Feixas et al., 2012) of the Clinical Outcomes in Routine Evaluation-Outcome Measure (CORE-OM) is used. It is a questionnaire specifically designed to evaluate the changes that occur in a therapeutic process of patients with various symptoms. It consists of 34 items completed by the patient and it includes four dimensions: (1) Well-being, four items that assess general well-being/malaise; (2) Problems/Symptoms, 12 items that assess anxiety, depression, stress and their somatic manifestations; (3) General functioning, 12 items that assess interpersonal and social relationships and functioning in activities of daily living; and (4) Risk, six items that assess attempts at self-harm, self-harm and aggressive behaviors directed at third parties; (5) HoNOS: The Health of the Nation Outcome Scales (HoNOS), designed by Wing et al. (1998) and translated into Spanish by Uriarte et al. (1999), is an instrument that consists of a set of scales designed to measure the whole range of physical, personal and social problems associated with mental illness and it can be used by Mental Health professionals routinely and in a clinical context. It is composed of 12 items and includes 4 subscales (behavioral problems, deterioration, clinical problems and social problems).

### Procedure

All the subjects filled out the SOFTA-o and the CORE-OM before the intervention began and refilled them once it was concluded. Therapists did the same with the HoNOS.

The interventions lasted between 15 and 20 sessions (45 min per session). The subjects themselves chose whether they wanted to be treated face-to-face or online, because this is a naturalistic observation study and we wanted to know if there was a preference for either of the two modalities. The online interventions were carried out through videoconference.

All subjects were treated by the same therapist (who made the diagnoses guided by their clinical judgment). This therapist is a male psychologist with a PhD in psychology, accredited training in psychotherapy, and over 10 years of experience.

The subjects filled out the questionnaires individually and independently, and they were only assisted by the researcher only when requested.

### Ethics approval

The study was approved by the Research Ethics Committee of the Vidal i Barraquer Mental Health University Institute.

## Results

### Description of analyses

The statistical analyses were conducted using the SPSS statistical package (version 29.0.0.0(241), SPSS Inc). Firstly, the descriptive results of demographic data, the diagnosis, the TA, CORE-OM and HoNOS were presented. Subsequently, the relation between CORE-OM and HoNOS with intervention modality, gender, age, diagnosis and TA were presented. Afterwards, the mixed model analysis was conducted. To calculate it, an unstructured variance–covariance matrix was computed via the restricted estimation of maximum likelihood. The CORE-OM and HoNOS before and after treatment, treatment modality, and their interactions were considered fixed effects. Finally, TA, gender and age were also included as fixed factors. The random effect was the subjects’ intersection parameter. The degrees of freedom were calculated with the Satterthwaite approximation. The end model was chosen by recalculating the models with and without interaction via maximum likelihood in order to compare the significance of the change on the Akaike information criterion (AIC). The residuals of prediction and of the random factor were inspected via a quartile-quartile plot to assess the suitability of the model.

### Descriptive results of the sociodemographic data, diagnosis, therapeutic alliance, intervention modality, CORE-OM and HoNOS

As seen on Table 1, the percentage of men and women was quite similar, with 81 (43.3%) men and 106 women (56.7%). The mean age was 23.01 (SD 2.83) years old. The 46% (*n* = 86) chose online modality, while the 54% (*n* = 101) opted for the face-to-face modality. Therefore, although the distribution is quite similar, there is a certain preference for the face-to-face modality.

The most prevalent diagnosis was anxiety (*n* = 58.30%), followed by depression (*n* = 20, 10.7%) and abuse (*n* = 28.15%).

Regarding TA scores, the SOFTA-o pre-intervention score is 8.58 (SD 3.87) and the post-intervention score is 36.11 (SD 5.34).

As for the CORE-OM scores, it is seen that the mean in the pre-intervention administration was 108 (SD 7.2) and, instead, post-intervention was 75.7 (SD 5.12).

Lastly, the mean HoNOS scores show that, in the pre-intervention administration, the result is 39.65 (SD 3.43), while in the post-intervention administration is 22.68 (SD 2.89).

### Comparison between age, sex, modality, diagnosis and TA in relation to the CORE-OM and HoNOS score before and after treatment

We conducted, with an IC 95%, a *t* test for the variables sex and modality; we used the Pearson correlation coefficient for age, TA, CORE-OM and HoNOS and ANOVA for the diagnosis.

Via the Pearson correlation coefficient, as seen in Table 2, in the CORE-OM there is no correlation between the two moments of administration of the instrument (*r* = 0.057, *p* = 0.437). Likewise, there is no correlation in the HoNOS between the two moments of administration of the questionnaire (*r* = −0.011, *p* = 0.877).

**Table 2:** CORE-OM, HoNOS, TA and age correlation before and after intervention.

Correlation	Value *r*	*p-*value	HoNOS pre *r* (P) value	HoNOS post *r* (P) value	AT pre *r* (P) value	AT post *r* (P) value	Age *r* (P) value
CORE-OM before treatment	0.057	0.437	0.195 (0.007)	−0.047 (0.522)	0.047 (0.520)	0.087 (0.234)	−0.042 (0.572)
CORE-OM after treatment	0.057	0.437	–0.087 (0.239)	0.447 (<0.001)	0.018 (0.810)	–0.471 (<0.001)	–0.106 (0.150)

Correlation	Value *r*	*p-*value	**CORE-OM pre** ***r* (P) value**	**CORE-OM post** ***r* (P) value**	AT pre *r* (P) value	AT post *r* (P) value	Age *r* (P) value
HoNOS before treatment	–0.011	0.877	0.195 (0.007)	–0.087 (0.234)	0.120 (0.101)	0.084 (0.228)	0.048 (0.510)
HoNOS after treatment	–0.011	0.877	–0.047 (0.522)	0.447 (<0.001)	–0.054 (0.422)	–0.882 (<0.001)	0.035 (0.633)

Regarding the CORE-OM scores before treatment, it is shown a significant correlation with HoNOS before treatment (*r* = 0.195, *p* = 0.007). And, in reference to the CORE-OM scores post-treatment, there are significant correlations with HoNOS post-treatment (*r* = 0.447, *p* < 0.001) and the TA post-treatment (*r* = −0.471, *P* = <0.001). The same happens with post-treatment HoNOS scores and post-treatment TA scores (*r* = −0.882, *p* < 0.001). These results suggest that patients’ scores on CORE-OM and therapist’s scores on HoNOS are related, meaning there is a relation in the change perceived by therapists and the patients between the pre and post treatment. Even so, therapists perceive a greater improvement after treatment than patients do. Furthermore, it is observed that the CORE-OM and HoNOS scores after treatment (i.e., an improvement in symptomatology) are related to a better TA after treatment.

As seen on Table 3, the *t-*test for independent samples revealed that there are no significant differences between the CORE-OM before treatment, neither in relation to the modality (*t* = −0.310, *p* = 0.378, *d* = 0.15) nor with the gender (*t* = −0.182, *p* = 0.428, *d =* 0.12). Regarding the CORE-OM scores after treatment, it is shown there is no significant relation with gender (*t* = −1′605, *p* = 0.055, *d =* 0.10) but there is with modality (*t* = 7.382, *p* < 0.001, *d* = 0.66). As for HoNOS, there are no difference between the scores before the intervention in neither modality (*t* = −0.847, *p* = 0.199, *d* = 0.16) nor gender (*t* = 0.464, *p* = 0.322, *d* = 0.04). On the other hand, HoNOS scores after treatment show that there is a significant relation with modality (*t* = 22.923, *p* < 0.001, *d =* 0.91) but not with gender (*t* = −10.373, *p* = 0.086, *d* = 0.07). Therefore, we can conclude that what both patients and therapists report is that face-to-face modality obtains better results than the online modality.

**Table 3:** CORE-OM and HoNOS comparison between modality and gender before and after intervention.

Test	Value	df	*P-*value	Effect size
CORE-OM before				
Modality	–0.310	185	0.378	0.15
Gender	−0.182	185	0.428	0.12
CORE-OM after				
Modality	7.302	185	<0.001	0.66
Gender	−1.605	185	0.055	0.10
HoNOS before				
Modality	−0.847	185	0.199	0.16
Gender	0.464	185	0.322	0.04
HoNOS after				
Modality	22.923	185	<0.001	0.91
Gender	−1.373	185	0.086	0.07

Finally, in relation to the diagnosis, an ANOVA was performed to determine whether there were any differences in the CORE-OM and HoNOS scores by diagnosis, and the results before and after treatment for the two instruments showed non-significant differences. As seen in Table 4, the results for the CORE-OM before and after treatment are *F* = 2.626, *p* = 0.170 and *F* = 3.306, *p* = 0.278, respectively; and for the HoNOS, *F* = 1.843, *p* = 0.194 and *F* = 2.117, *p* = 0.446 for before and after the treatment, respectively.

**Table 4:** CORE-OM and HoNOS comparison by diagnosis.

ANOVA	Diagnosis mean square	*f*	*P-*value
CORE-OM before treatment	2917.107	2.624	0.170
CORE-OM after treatment	3381.538	3.306	0.278
HoNOS before treatment	219.244	1.843	0.194
HoNOS after treatment	918.678	2.117	0.446

In this case, when comparing the effect size between the variables, it was observed that it was small in all the pairings, since no comparison showed an effect size greater than *d* = 0.20.

### Analysis of the mixed model

We performed an analysis for CORE-OM and another for HoNOS separately.

Firstly, in the model without interactions, with IC 95%, the pre-post change in the CORE-OM was significant (*t*₃₇₀ = −38.870, *p* < 0.001, *d* = 0.83), as was the treatment modality (*t*₃₇₀ = −22.483, *p* = 0.003, *d =* 0.69). Age, gender, and TA did not reach the level of significance (*t*₃₇₀ = 0.204, *p* = 0.87, *d =* 0.11; *t*₃₇₀ = −0.098, *p* = 0.72, *d* = 0.13; *t*₃₇₀ = −3.344, *p* = 0.072, *d* = 0.14 respectively).

The model with interactions (AIC = 2784.4, with 12 parameters), with IC 95%, was significantly better (X^2^₄ = 823.42, *p* < 0.001) than the model without interactions (AIC = 3845.2, with 8 parameters). The interaction between the time of the evaluation and the therapeutic modality was highly significant (*t*₁₈₃ = −36.974, *p* < 0.001, *d =* 0.91). In the web-based treatment, the mean score on CORE-OM decreased 11.24 points (SD 3) while in the face-to-face treatment, it decreased 29.12 (SD 5.2). The rest of the interactions were not significant. In the inspection of the residuals, no gross deviations were found compared to a normal distribution.

In the HoNOS analysis, in the model without interactions, with IC 95%, the pre-post change in the HoNOS was significant (*t*₃₇₀ = −42.637, *p* < 0.001, *d* = 0.88), as was the treatment modality (*t*₃₇₀ = −31.354, *p* < 0.001, *d =* 0.86). Age, gender, and TA did not reach the level of significance (*t*₃₇₀ = −1.754, *p* = 0.554, *d =* 0.6; *t*₃₇₀ = −1.012, *p* = 0.487, *d* = 0.5; *t*₃₇₀ = −2.876, *p* = 0.112, *d =* 0.09, respectively).

The model with interactions (AIC = 2372.6, with 12 parameters), with IC 95%, was significantly better (*X*^2^₄ = 745.22, *p* < 0.001) than the model without interactions (AIC = 4056.1, with 8 parameters). The interaction between the time of the evaluation and the therapeutic modality was highly significant (*t*₁₈₃ = −43.842, *p* < 0.001, *d* = 0.89). In the web-based treatment, the mean score on HoNOS decreased 7.89 points (SD 3.1) while in the face-to-face treatment, it decreased 15.2 (SD 4.2). The rest of the interactions were not significant. In the inspection of the residuals, no gross deviations were found compared to a normal distribution.

## Discussion

In accordance with what we pointed out in the introduction, we see that the results indicate a current trend that seems to be going in the direction of a greater preference for online intervention over face-to-face (Yılmaz et al., 2024), when the choice is free and there are no conditions that dictate the choice towards one of the two modalities (geographical distance, technical difficulties, suggestion by the therapist, etc.). In the case of our study, the slight increase in the face-to-face preference is especially significant when considering the average age of the participants (23 years), a social group in which the relationship through different devices at different levels of affective significance (partner, family, friends, acquaintances, strangers, etc.) is commonly present and, therefore, has been experienced and incorporated with its advantages and limitations. At the same time, given the characteristics of the sample (university students), it is reasonable to think that they have sufficient technological resources and personal skills for the experience they have in the two media in which they develop their relationships (face-to-face and online) to be sufficiently normalized and comfortable, which means that their preference is probably more related to other personal factors (need for direct contact with the other, or need to differentiate the therapeutic relationship from others).

It is likely, as happens with so many other resources that promote the qualities of “speed and convenience,” once the first periods of fascination and idealization (“technological infatuation”) have passed, that this human group is in a new phase in which preferences are distributed in the proportion that appears in our study or that they may even be equal (50%) but that they stabilize at the level of the general population in similar conditions.

On the other hand, the results suggest that the patients’ scores on the CORE-OM and the therapists’ scores on the HoNOS are related, indicating that there is a relationship in the change perceived by both between before and after the intervention. However, the therapists perceive a greater improvement after the treatment than the patients. In addition, it is observed that the CORE-OM and HoNOS scores after the treatment (that is, an improvement in the symptomatology) are related to a greater TA after the treatment. Thus, according to Norcross and Lambert (2011, 2019), among all the factors that affect in one way or another, the therapeutic change, the alliance between therapist and patient is confirmed as the most decisive predictive element. Furthermore, in the study, the coincidence of this perception in both the therapist and the patient is especially important for the objectives of the research, since it offers a perspective that gives depth and content to the results of a reduction in symptomatology. This could, in fact, occur without being accompanied by a particularly significant therapeutic alliance, as is the case with many less systematized therapeutic interventions than psychotherapy or from other orientations in which suggestive techniques prevail, for example, as Leuchtenberg et al. (2022) pointed out.

That therapists perceive a more pronounced improvement at the end of the treatment is consistent with the intervention modality that has been established in this study. In treatments that we consider brief and/or focal, the psychotherapist’s perception encompasses elements of assessment that “yet” have not yielded results and therefore are not perceived in the same way by the patient. Aspects such as increased insight or the flexibility of defensive mechanisms can be observed quite precisely by the therapist during the course of treatment, but they require the passage of time (accompanied by the patient’s life experiences) for them to hatch and translate into what we know as therapeutic change (this is why measurements of change 6 months after finishing treatment or a year later, for example, are important). Online therapy can present challenges related to limited emotional depth, personalization and ethical considerations, which can affect essential elements for psychotherapy effectiveness such as empathy and later on influence the quality of therapeutic alliance (Malouin-Lachance et al., 2025).

Another important result of the study is that in the CORE-OM post-intervention and HoNOS post-intervention a significant relationship is observed with the intervention modality, concluding that what both patient and therapist report is that the face-to-face modality obtains better results than the online one. That the face-to-face modality is better valued than the online one is in accordance with the preliminary studies carried out by the authors (Mercadal and Cabré, 2022). Once again, it is particularly important that there is agreement between the therapists’ assessment and the patients’ assessment. In the case of our study, in addition, we believe it is important to highlight that the therapist (by age, he is 33 years old) does not have the bias that could lead to a preference for face-to-face treatment. An older therapist who had always practiced face-to-face treatment and who had started more recently in the online modality, could tend to value the former better, as a result of his experience. And therapists who feel capable of providing empathy and support in online settings are the ones who have previously used this technique (Sperandeo et al., 2021).

Finally, the results of the mixed models confirm what we have discussed so far. And, furthermore, they add that the interaction between modality and time correlate very significantly in both the CORE-OM and the HoNOS, showing that the face-to-face modality is more effective than the online one.

Even so, we believe that for future research we have to solve some limitations, such as extending the age range of the sample, administering the questionnaires 6 months after the end of the intervention, increasing the number of therapists, and focusing more on their characteristics to study them as another variable that may affect therapeutic results. In this regard, it would also be important to include therapists with different therapeutic orientations, which would enrich the scope of the study and the explanatory capacity of its findings. Likewise, it would be useful to include therapists with different levels of experience, as this variable seems to be associated with improvements in the therapeutic alliance (Shelef et al., 2005).

## Data Availability

The raw data supporting the conclusions of this article will be made available by the authors, without undue reservation.
